# Supplementary material for: Parity transitions in the superconducting ground state of hybrid InSb–Al Coulomb islands
Source: Nat Commun. 2018 Nov 15;9:4801. doi: 10.1038/s41467-018-07279-7 (PMC6237907; doi:10.1038/s41467-018-07279-7)
Supplement: Supplementary file 1 — Supplementary Information [file 41467_2018_7279_MOESM1_ESM.docx]

**Supplementary Note 1. Growth of epitaxial InSb/Al nanowire islands**


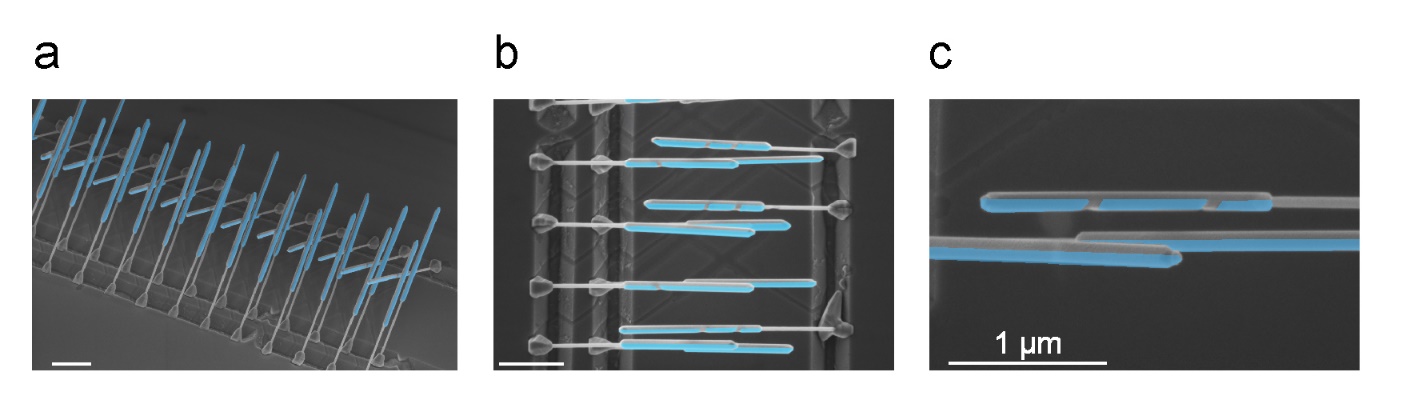


**Supplementary Figure 1. Scanning electron microscope (SEM) image of shadowed InSb/Al nanowires.** The Al shell on the nanowires is coloured in light blue. **a**, InSb nanowires are grown in a metal-organic vapour-phase epitaxy (MOVPE) reactor from catalyst droplets (Au), positioned along etched trenches. First, InP stems are grown to facilitate the nucleation of InSb nanowires. After nanowire growth, hydrogen cleaning is used to remove the native oxide layer of the nanowires and Al is evaporated in a direction parallel to the trenches. The positioning of Au droplets together with the tilting of the Al evaporation direction with respect to the horizontal plane allows the shadowing of predefined sections on the nanowires. **b**, Zoomed-in SEM image of InSb nanowires with either one or two shadows. **c**, During the Al evaporation, two sacrificial nanowires at the bottom are used to shadow two short segments ($\sim$100 nm) on a third wire at the top. The island length, set by the separation between two shadows, varies between 0.2 and 1 μm. Note that this ‘shadow-growth’ mechanism avoids the need of etching the Al, leaving pristine nanowire facets on the junction regions. We create a hybrid superconducting-semiconductor island of Al-InSb by interrupting the Al shell in two narrow regions allowing local electrostatic gating of two semiconducting junctions. At these two regions tunnel barriers can be introduced by the top gates to confine the superconducting-semiconducting hybrid island.

**Supplementary Note 2. Extracting *E*_c_ and** ***E*_0_ from 2*e*-periodic Coulomb diamonds at zero magnetic field**

The energy parabolas in Supplementary Figure.2a illustrate that the degeneracies of the even-parity ground-state parabolas occur at *E*_c_. In the Coulomb valley (at *N*_g_ = 0) the even-parity parabolas cross at 4*E*_c_ (indicated by the yellow dot in Supplementary Figure.2a). In finite-bias Coulomb diamonds, the voltage drop at the top of the 2*e*-periodic Coulomb diamonds corresponds to 8*E*_c_/*e* (dashed yellow diamond in Supplementary Figure.2b). As a result, *E*_c_ extracted from the yellow-dashed diamond in Fig. 1b of the main text is $\sim$22 μeV. Over the entire gate range *E*_c_ varies between 22-27 μeV (see typical diamonds in Supplementary Figure.2c and d at different gate values).

Above the degeneracy points of the GSs, the onset of quasiparticle transport causes a blockade of Andreev reflection and results in a region of negative differential conductance (NDC) starting from a threshold voltage bias *V*_NDC_ ≈ 2(*E*_0_ - *E*_c_)/*e* (see the blue arrows in Supplementary Figure.2a) [2]. For example, *V*_NDC_ = 90 μV in Fig. 1b, so *E*_0_ ≈ 67 μeV. On the other hand, at finite bias, the onset of 1*e*-periodicity is due to single-particle transport via co-tunnelling events (see the red arrow in Supplementary Figure.2a), corresponding to 2*E*_0_/*e* [3]. In Fig. 1b, *E_0_* extracted from the onset of 1*e*-periodicity is close to the number extracted from NDC in the same diamond (*E*_0_ ≈ 67 μeV). Over the entire gate range *E*_0_ varies between 50 - 90 μeV (see diamonds in Supplementary Figure.2c and d).


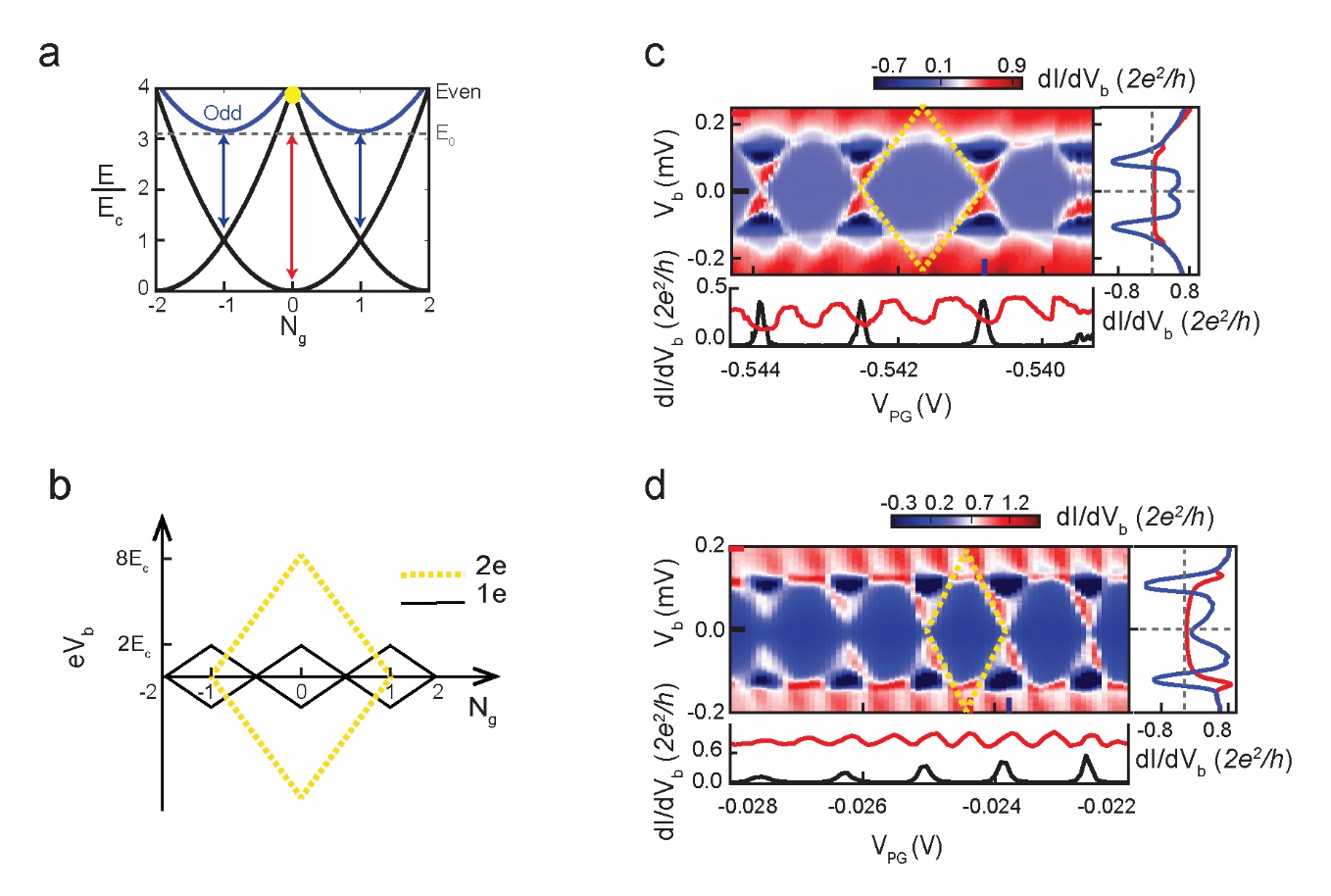


**Supplementary Figure 2. Derivation of *E*_c_ and *E_0_* from Coulomb diamonds. a**, The energy level parabolas of the superconducting island. The black parabolas describe the charge states for even parity, and blue parabolas for odd parity. Odd parabolas are lifted by *E*_0_, consistent with *E*_0_ $\gg$ *E*_c_ in Fig. 1b. **b**, Finite-bias Coulomb diamonds for 1*e* (solid black diamonds) and 2*e*-periodicity (dashed yellow diamond). **c** and **d**, Coulomb diamonds for two different gate configurations. In the bottom panels, horizontal linecuts show the 2*e* (in black) versus 1*e*-periodic (in red) conductance oscillations taken respectively at *V*_b_ = 0 μV and *V*_b_ = 250 μV (in **c**) / *V*_b_ = 200 μV (in **d**). In the right panels, vertical linecuts at different *V*_PG_ voltages show the presence of NDC regions above the degeneracy point (blue linecuts) and conductance enhancement in the valley (red linecuts). For **c**, we estimate *E*_c_ ≈ 25 μeV, while the onset of NDC is found at *V*_b_(NDC) ≈ 70 μV, so *E*_0_ ≈ 60 μeV. In **d**, *E*_c_ ≈ 22 μeV and *V*_b_(NDC) ≈ 70 μV, so *E*_0_ ≈ 57 μeV.

**Supplementary Note 3. Superconducting critical magnetic fields of the InSb/Al nanowire for three orientations**

We performed tunnelling spectroscopy at one of the two junctions as a function of the magnetic field strength in three directions: parallel to the nanowire $B_{\text{∥}}$ (Supplementary Figure.3a), perpendicular to the substrate $B_{\text{out of plane}}$ (Supplementary Figure.3b) and perpendicular to the nanowire in the plane of the substrate $B_{\text{in plane}}$ (Supplementary Figure.3c). The local tunnel gate voltage is -1 V for this spectroscopy junction (the weak-tunnelling regime). The voltages are +2 V for the other tunnel gate and the plunger gate to make sure the chemical potential is smooth for the entire island except at the local tunnel gate. When *B* is applied parallel to the nanowire (Supplementary Figure.3a), the hard superconducting gap persists up to 1.0 T, with Δ($B_{\text{∥}}$ = 0) = 220 μeV and Δ($B_{\text{∥}}$ = 0.8 T) = 90 μeV (the bottom panel in Supplementary Figure.3a). The gap closes completely at $B_{\parallel}^{c}\geq$ 1 T, which is out of range for the employed 3D vector magnet.

For the other two orientations, our device undergoes a transition to the normal state at $B_{\text{out of plane}}^{\text{c}} \approx$ 0.12 T and $B_{\text{in plane}}^{\text{c}} \approx$ 0.18 T. In the main text, $B_{\text{⊥}}$ means $B_{\text{in plane}}$ for simplicity. This observation is consistent with the SEM images of the device in Fig. 1a, showing the Al shell covering the top facet and one of the side facets of the nanowire.


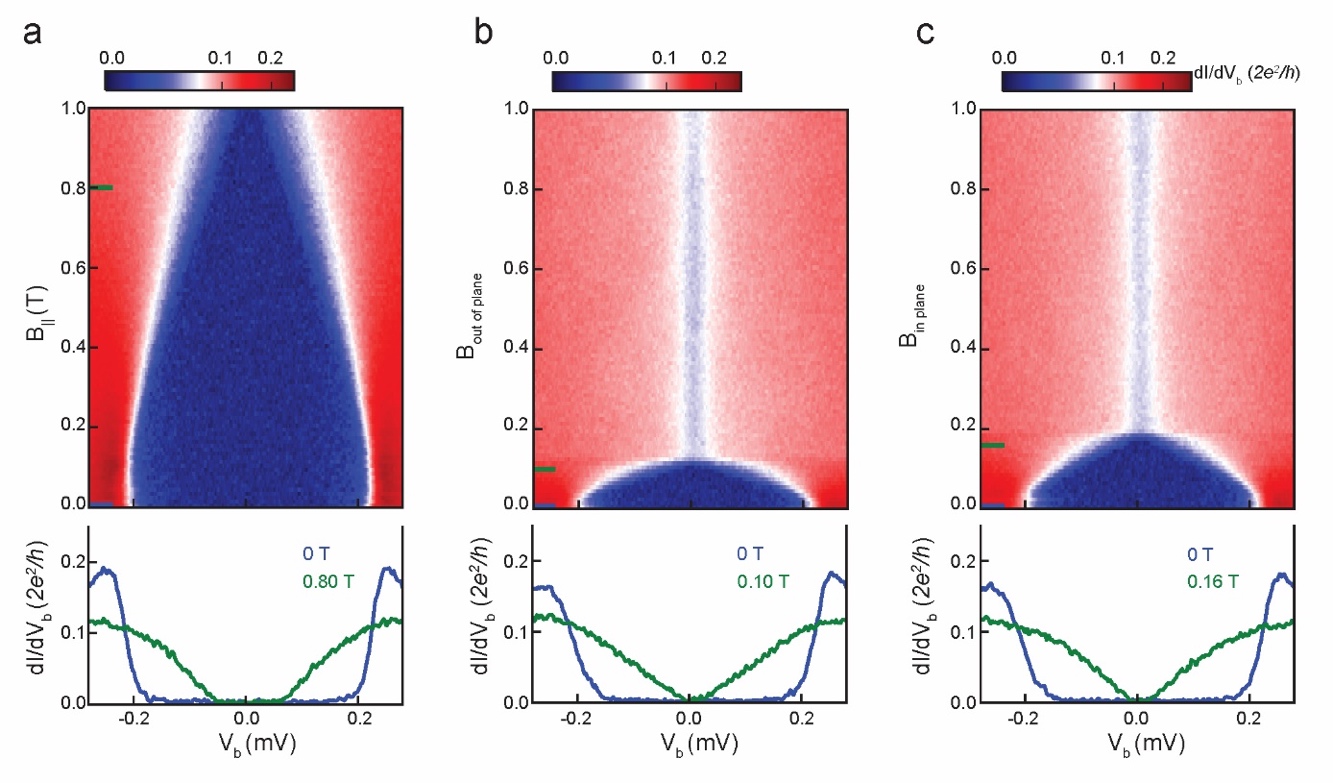


**Supplementary Figure 3. Tunnelling spectroscopy at magnetic fields of different orientations.** Top panels show d*I*/d*V*_b_ tunnelling spectroscopy as a function of $B_{\text{∥}}$ (**a**), $B_{\text{out of plane}}$ (**b**) and $B_{\text{in plane}}$ (**c**). Typical linecuts at selected *B* are shown in the bottom panels. The zero-bias dip above the critical magnet field in **b** and **c** are likely due to the confinement at the second junction.

**Supplementary Note 4. Raw data for d*I*/d*V_b_* as a function of *V_PG_* and** $\boldsymbol{B}_{\boldsymbol{\parallel}}$


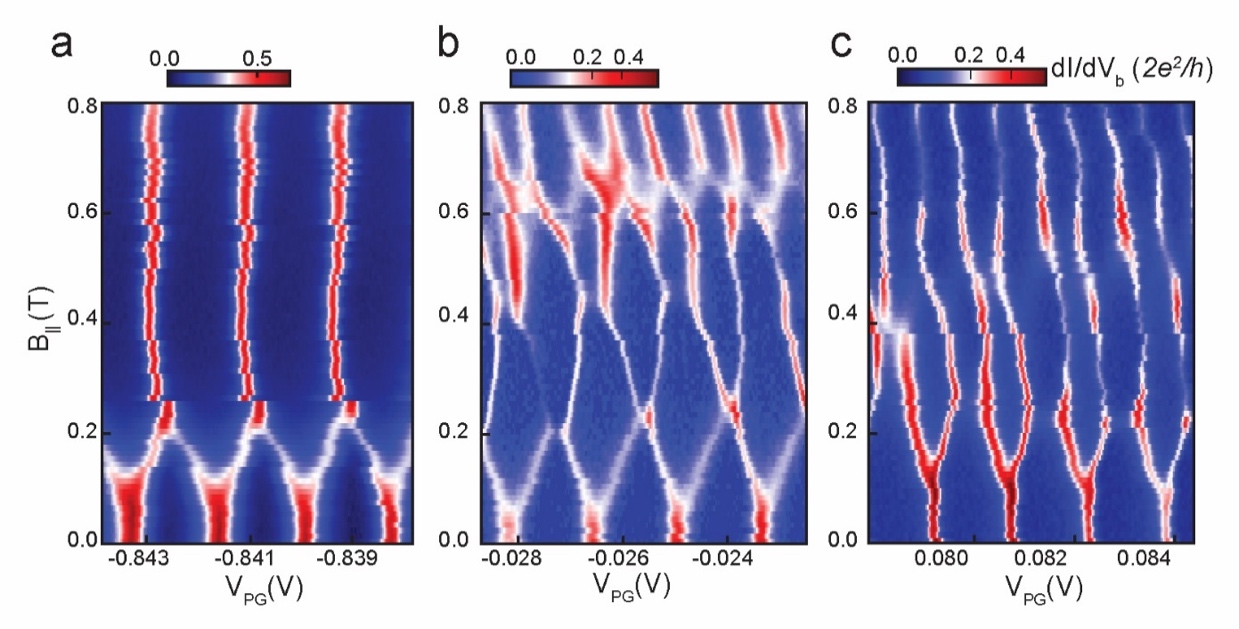


**Supplementary Figure 4. Raw data for d*I*/d*V*_b_ as a function of *V_PG_* and** $\boldsymbol{B}_{\boldsymbol{\parallel}}$**. a**, **b**, and **c** correspond to Fig. 2b, Fig. 4b and Fig. 4d in the main text, respectively. The plunger gate is sometimes drifting and gate-voltage jumps can occur because of charge trapping in the dielectric, so that raw dI/dV_b_ data here are not always stable. However, the conductance peaks from different parity states are still easily identified. A few common offsets in VPG are introduced to compensate for the shifts in gate voltage.

**Supplementary Note 5. Comparison of the 2e-periodic odd-parity ground state with previous observations of fermion parity crossings**

The ground state fermion parity of a superconducting system can be changed by the Fermi-level crossing of a spin-resolved subgap state. The subgap state causing the GS transition may, for instance, be bound to an impurity in a bulk superconductor, as in the case of Shiba-Yu-Rusinov states [4]; or it may be an Andreev bound state in a Josephson junction (see [5] for instance). In our case, the fermion parity switch is due to a subgap state localized in the mesoscopic InSb/Al superconducting island. In this case, as discussed in the main text, when the energy gap for emptying the subgap state exceeds the charging energy of the island, the odd parity GS caused by the fermion parity switch is stable at all value of the induced charge of the island and the sequential process is Cooper pair tunnelling/Andreev reflection. This condition was not reached in previous studies of hybrid superconducting-semiconductor islands, which always observed either an even-parity GS or an alternation of even- and odd-parity GS [6, 7]. Furthermore, our observation is also distinct from the parity transitions observed in semiconducting quantum dots proximitized by a superconducting lead [8], where the charging energy applies only to the semiconductor but not to the superconductor. In this case, reducing the strength of the proximity effect (by varying the coupling between the dot and the superconductor) may cause a change from even to odd parity in the ground state of the dot, but only at values of the induced charge which would favour an odd occupation of the dot in the absence of the superconducting lead. In other words, the effect does not require a change of the GS parity of the superconducting lead itself.

**Supplementary Note 6. Effect of parallel and perpendicular magnetic fields on the periodicity of Coulomb peaks**

For Figs. 2c and 4d of the main text, we just show typical Coulomb diamonds (Fig. 4f) at a specific value of $B_{\text{∥}}$. The additional figures at different $B_{\text{∥}}$ and $B_{\text{⊥}}$ (presented in Supplementary Figure.6) demonstrate the robustness of the isolated zero-bias state at all $B_{\text{∥}}$, as well as the obvious difference between the superconducting state at $B_{\text{∥}}$ and the normal state at $B_{\text{⊥}}$. Supplementary Figures.6a-c correspond to the same gate settings as Fig. 4d and Supplementary Figure.6d relates to Figs. 2c-d. The diamonds at different $B_{\text{∥}}$ (Supplementary Figure.6b and the top two panels in Supplementary Figure.6d) show the consistence of the isolated zero-mode. The finite bias spectroscopy (Supplementary Figure.6a) at the degeneracy point of Fig. 4d, as well as Supplementary Figure.6b, proves there is a zero-energy crossing at low $B_{\text{∥}}$ and a sticking zero-energy state at high $B_{\text{∥}}$, which fits the sketch in Fig. 4a. The normal transition in Supplementary Figure.6c shows equal peak spacings and heights, which is used to distinguish the normal and superconducting 1*e*-periodic Coulomb peaks. For figs. 2c and d, we can also see the isolated zero-mode for different $B_{\text{∥}}$ and a continuum in the normal regime (Supplementary Figure.6d).

*
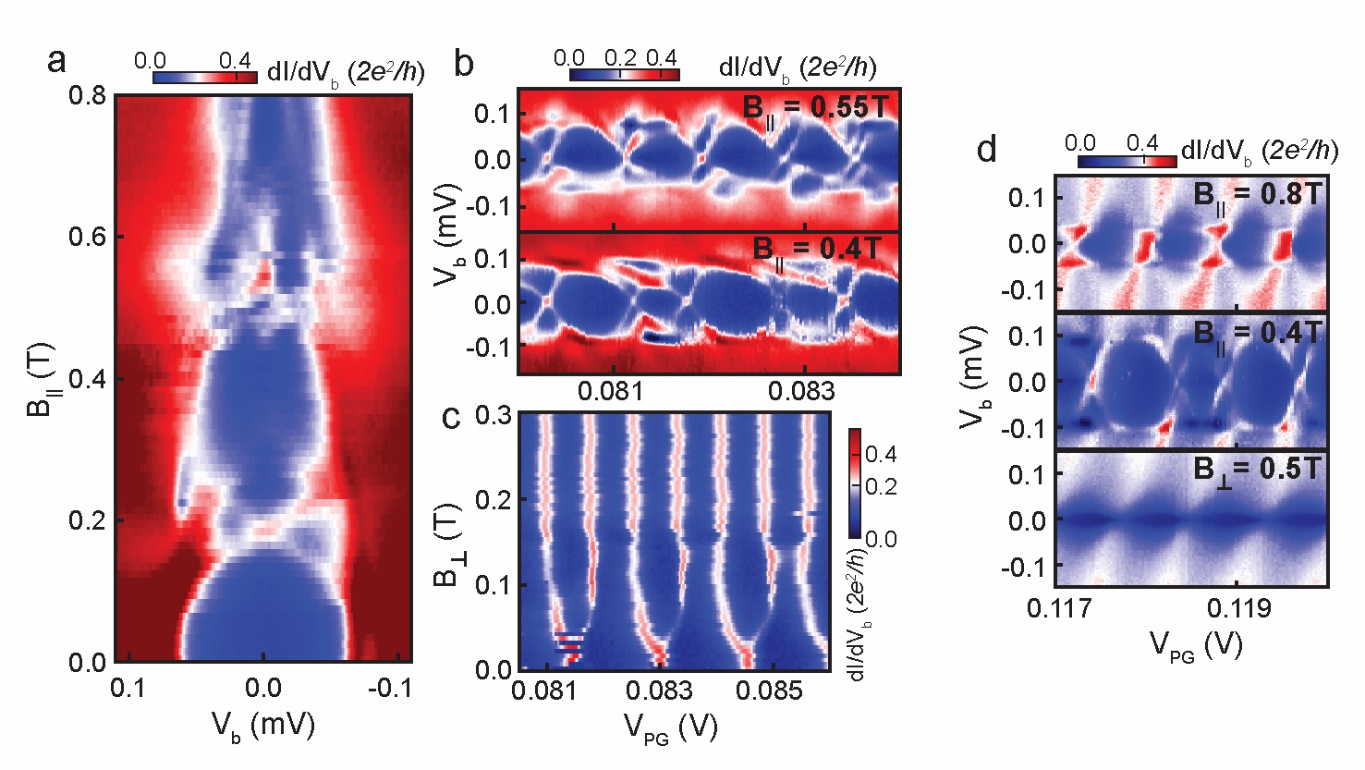
*

**Supplementary Figure 6. Zero-bias resonances at different** $\boldsymbol{B}_{\text{∥}}$ **and** $\boldsymbol{B}_{\text{⊥}}$**.** **a**, **b** and **c** are measured for a similar gate-voltage regime as Fig. 4d. **a**, d*I*/d*V*_b_ as a function of *V*_b_ and $B_{\text{∥}}$ at *V_PG_* close to one degeneracy point in Fig. 4d. A charge degeneracy point at first crosses the Fermi level at $B_{\text{∥}}\approx$ 0.2 T, while a more persistent zero-bias peak occurs for $B_{\text{∥}}$ = 0.5 - 0.65 T. **b**, Coulomb diamonds at $B_{\text{∥}}\approx$ 0.4 T and 0.55 T. Both of them show a discrete state at the degeneracy points, isolated by an energy gap. $G_{\text{e}\text{→}\text{o}}$ and $G_{\text{o}\text{→}\text{e}}$ also show alternating amplitudes. **c**, Evolution of zero-bias conductance peaks with $B_{\text{⊥}}$. The state becomes normal at $B_{\text{⊥}}\approx$ 0.18T ($B_{\text{in plane}}^{\text{c}}$ in Fig. S3c), and the peak oscillations become 1*e*-periodic with equal peak heights. **d**, Coulomb diamonds at different $B_{\text{∥}}$ and $B_{\text{⊥}}$ at the same gate-voltage regime as Figs. 2c and 2d. Both of the top and middle panels at finite $B_{\text{∥}}$ show a discrete level at the degeneracy points and alternating peak heights, whereas the diamonds in the normal regime in the bottom panel show normal 1*e* oscillations without isolated peaks at the charge degeneracy points.

**Supplementary Note 7. Fitting of the Coulomb resonances**

The Coulomb resonances are analysed by fitting all peaks simultaneously using an identical electron temperature $T_{\text{el}}$ that takes into account the temperature-broadening of the Coulomb resonances. A single resonance is described by a Breit-Wigner distribution [9, 10]

$G_{\text{BW}} \left( V_{\text{PG}},V_{0},E \right)=\frac{2e^{2}}{h}\frac{\left( {h\Gamma}/2 \right)^{2}}{\left( {h\Gamma}/2 \right)^{2}+\left[ e\alpha\left( V_{\text{PG}}-V_{0} \right)-E \right]^{2}}$ (1)

where $V_{\text{0}}$ is the centre of the Coulomb peak and $\alpha$ is the plunger gate lever arm. The line shape is thermally broadened according to [11]

$G\left( V_{\text{PG}},V_{0} \right)\propto\int_{-\infty}^{\infty} G_{\text{BW}}\left( V_{\text{PG}},V_{0},E \right)\left[ -\frac{\partial f\left( T,E \right)}{\partial E} \right]dE$ (2)

with the Fermi-Dirac distribution $f\left( T,E \right)$. Hence, the total fitting function is given by a sum over a number of these line shapes and the tunnel coupling to the leads $\Gamma$. The peak position $V_{0}$ and the peak height are individual fitting parameters for each resonance, while the electron temperature $T_{\text{el}}$ and a constant offset are used as common fitting parameters.

In conclusion, by fitting the data as depicted exemplarily in Supplementary Figure.7a (blue) the fitted curve (green) describes the data very well and we find an electron temperature of about 20-50 mK and a typical tunnel coupling of about $h\Gamma=5 \mu eV$.

As depicted in Fig. 4b (bottom panel) of the main text the Coulomb peak spacing of the even and odd valleys oscillates as a function of magnetic field. The oscillation amplitude is strongly reduced above 0.6 T. In Fig. 4b also the relative Coulomb peak height $\Lambda$ is shown averaged for the three pairs of Coulomb peaks in Supplementary Figure.6b. This quantity is given by

$\Lambda=\frac{G_{\text{e}\text{→}\text{o}}}{G_{\text{e}\text{→}\text{o}}+G_{\text{o}\text{→}\text{e}}}$ (3)

where $G_{\text{e}\text{→}\text{o}}$ is the conductance peak height at resonance between an even and an odd parity Coulomb diamond and $G_{\text{o}\text{→}\text{e}}$ is the consecutive resonance between the odd and the next even parity Coulomb diamond [12]. Clearly, the relative peak height undergoes oscillations as well that extend also into the regime of stable 1*e* oscillations above 0.6 T. The fitting result for the data in Supplementary Figure.7b, which is used to extract the Coulomb peak spacings and peak heights, is depicted in Supplementary Figure.7c.

**
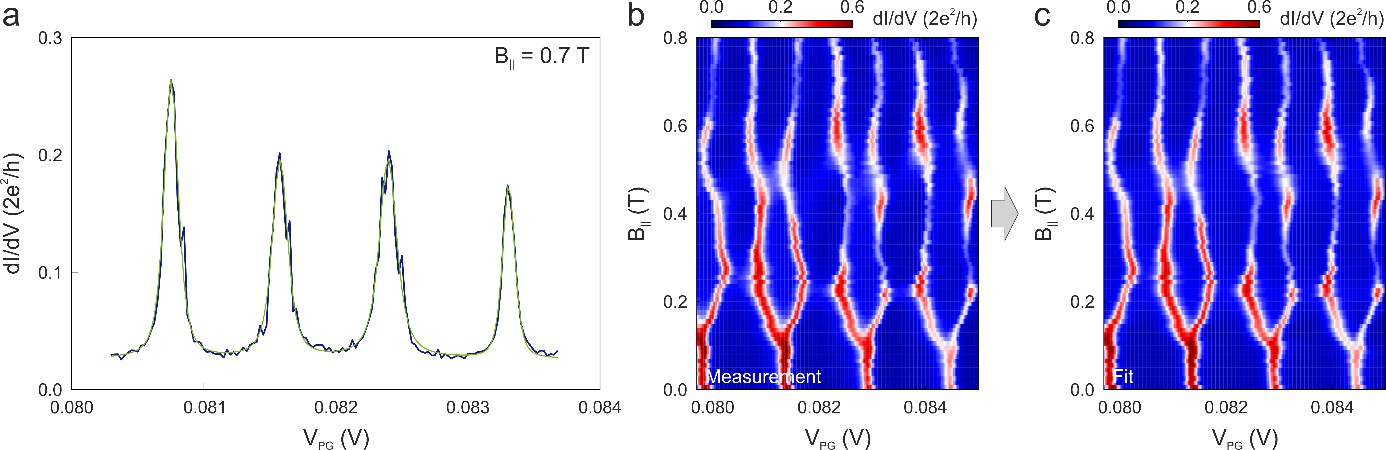
**

**Supplementary Figure 7. Fitting of the Coulomb resonances. a**, Fitting of the zero-bias linecut at $B_{\parallel}$ = 0.7 T from the data presented in Fig. 4d of the main text. **b** and **c**, Measurement (**b**) and fitting result (**c**) using $G_{\text{sum}}=\sum_{i} G\left( V_{\text{PG}},V_{0,i} \right)$, with $G\left( V_{\text{PG}},V_{0,i} \right)$ given by Supplementary Eq.(2), for the data presented in Fig. 4d of the main text.

**Supplementary Note 8. The relation between peak spacing and peak height ratio**

We observe that the oscillations in $\Lambda$ are similar in number and period to the corresponding oscillations in $S_{e}$ and $S_{o}$ (Supplementary Figure.8a, b and d), indeed suggesting a possible connection between the two.


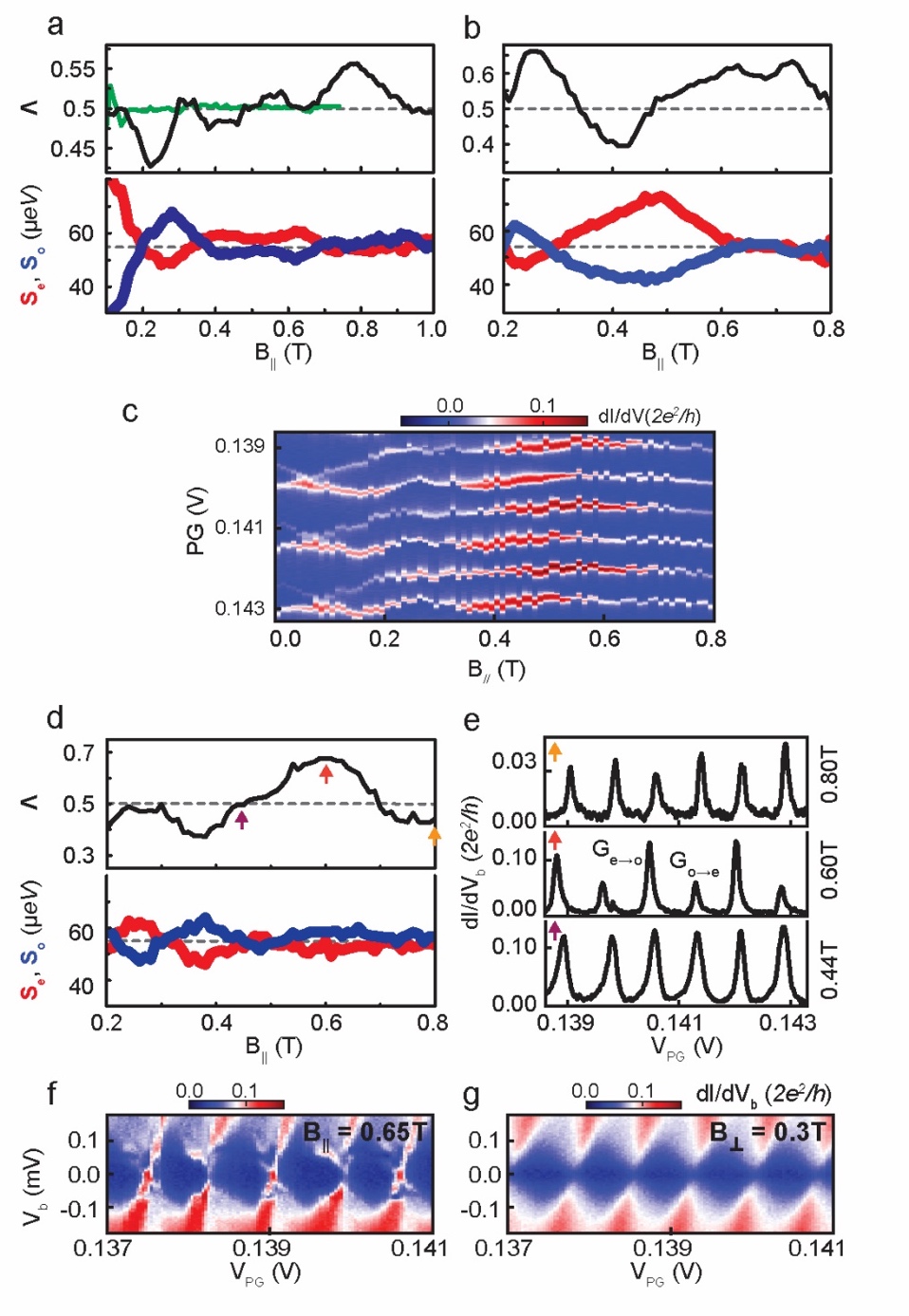


**Supplementary Figure 8. Evolution of peak heights and spacings.** Peak height ratio $\Lambda$ and peak pacings for even ($S_{e}$) and odd ($S_{o}$) parities as a function of $B_{\parallel}$ extracted from Fig. 2c (shown in **a**), Fig. 4d (shown in **b**) and Fig. S8c (shown in **d**). For comparison, the green curve in **a** is extracted from the normal state data of Fig. 2d. **e**, Exemplary Coulomb oscillations for the data in **c** at different fields $B_{\parallel}$ = 0.44 T ($\Lambda\approx$ 0.5), 0.60 T ($\Lambda$ > 0.5), and 0.80 T ($\Lambda$ < 0.5) indicated by arrows. **f**, Coulomb diamonds at a large value of $\Lambda$ in **c**, indicating the isolated zero mode. **g**, Coulomb diamonds in the same gate-voltage regime as **f**, but measured in the normal state ($B_{\perp}$= 0.3 T), showing the continuum at finite bias.

**Supplementary Information References**

1. Gazibegovic, S. *et al*. Epitaxy of advanced nanowire quantum devices. Nature 548, 434438 (2017).
2. Hekking, F. W. J., Glazman, L. I., Matveev, K. A. & Shekhter, R. I. Coulomb blockade of two-electron tunneling. Phys. Rev. Lett. 70, 4138 (1993).
3. Nazarov, Y. V., & Blanter, Y. M. Quantum transport: introduction to nanoscience. Cambridge University Press (2009).
4. Balatsky, A. V., Vekhter, I., and Zhu, J. X. Impurity-induced states in conventional and unconventional superconductors, Rev. Mod. Phys. 78, 373 (2006).
5. Chang, W., Manucharyan, V. E., Jespersen, T. S., Nygard, J. and Marcus, C. M. Phys. Rev. Lett. 110, 217005 (2013).
6. Higginbotham, A. P. *et al*. Parity lifetime of bound states in a proximitized semiconductor nanowire. Nat. Phys. 11, 1017 (2015).
7. Albrecht, S. M. *et al*. Exponential protection of zero modes in Majorana islands. Nature 531, 206 (2016).
8. Lee, E. J. H. *et al*. Spin-resolved Andreev levels and parity crossings in hybrid superconductor-semiconductor nanostructures. Nat. Nanotech. 9, 79 (2014).
9. Breit, G. & Wigner, E. Capture of slow neutrons. Phys. Rev. 49, 519 (1936).
10. Beenakker, C. W. J. Theory of Coulomb-blockade oscillations in the conductance of a quantum dot. Phys. Rev. B 44, 1646 (1991).
11. Foxman, E. B. *et al*. Effects of quantum levels on transport through a Coulomb island. Phys. Rev. B 47, 10020 (1993).
12. Hansen, E. B., Danon, J., & Flensberg, K. Probing electron-hole components of subgap states in Coulomb blockaded Majorana islands. Phys. Rev. B 97, 041411 (2018).
